# Three Cases of Nerve-Injury—Operation—Recovery

**Published:** 1888-04

**Authors:** H. H. Frothingham

**Affiliations:** 4306 Lake Avenue


### THREE CASES OF NERVE-INJURY. RECOVERY WITH OPERATION.

BY H. H. FROTHINGHAM, M. D.

[Read before the Chicago Medical Society, March 19, 1888.]

The first case was that of a young man, 20 years of age, who had received a razor-cut across the anterior surface of the arm, about four inches above the elbow-joint. Upon examination it was found that the cut extended to the humerus, dividing the brachial artery, median nerve, and partially severing the biceps and brachialis anticus muscles.

Under ether the artery was ligated at both ends, the muscles sutured with catgut, and the wound closed. An antiseptic dressing was applied, and over this a plaster cast fixing the arm in a flexed position, in order to remove all strain from the muscle sutures.

Twenty-four hours later the operator, becoming uneasy about the divided nerve, reopened the wound and sutured the divided sheath, thus approximating the nerve ends; the material used for suture being fine catgut, and the point so taken that the portion of the stitch contained within the nerve-sheath lay parallel to the nerve-fibres. A bone drainage-tube was introduced at the inner angle of the wound, which was closed and dressed as before.

For about twenty-four hours the patient complained of tingling in the first three fingers of the hand, after which time there was no discomfort. Sensation returned rapidly to the area supplied by the median nerve, being apparently normal at the end of five days.

Flexion of the fingers and thumb and of the hand upon the forearm, was possible after four days, and increased rapidly in extent.

The wound healed throughout by first intention, and was entirely well in twelve days. At this time, sensation in the hand and forearm appeared normal; flexion of the wrist and fingers was easily accomplished, but the grasp of the hand was weak.

Control over the muscles increased, and at the end of three weeks there was apparently normal innervation of the hand and forearm.

CASE II.—Male, aged 30. Injury: A razor-cut across the forearm about three inches above the wrist, severing the radial and ulnar arteries, median and ulnar nerves, and tendons of the flexors sublimis digitorium, carpi radialis, carpi ulnaris and palmaris longus. When first seen a compress was found placed over the forearm above the wound and drawn so tightly as to control hæmorrhage. The wound had been filled with Monsel's solution, and the skin incision stitched with superficial sutures of fine silk, leaving the ends of each thread a couple of inches in length. Cleansing and dressing of the wound involved the loss of some blood.

After thorough cleansing, the arteries were tied above and below, the nerves sutured as in Case I, and the severed tendons identified; the ends of each being then sutured with juniper catgut.

A catgut drain was placed deeply through the wound, the skin was sutured, a dressing applied, and the arm bound upon a Levis tin splint commonly used for fractured radius. Circulation was feeble, and the hand cold for two days, after which time the natural color and warmth were restored.

At this time sensation in the palmar surface of all the fingers and thumb was possible but slight.

Further examination into nerve condition was impossible at this time on account of the severed tendons.

Eight days after the injury the wound was entirely healed.

Flexion, abduction, and adduction of the thumb were possible, showing a return of function, at least in the median nerve.

Sensation in the hand was normal.

In four weeks the splint was removed and careful exercise advised. The patient had absolutely no pain, but a numb feeling in the hand lasting only for the first two days, and due probably to lack of blood-supply.

CASE III was that of a lady, aged about 35 years, who sustained a fracture of the right humerus by a fall upon one elbow.

The fracture was situated just below the insertion of the deltoid muscle, and was oblique; the displacement was extreme.

The patient complained of great pain in the forearm and hand, which was not relieved by reduction of the fragments of bone.

Morphine in large doses was necessary in order that any sleep might be obtained.

Two days later the pain was somewhat diminished and was referred by the patient to the area of distribution of the musculo-spiral nerve below the elbow.

Five days after the injury, motion of the wrist and fingers was seen to be impaired.

Seven days later the extensors and supinators of the hand and forearm were found to be completely paralyzed, and there was œdema of the back of the hand.

One month after the injury the condition was as follows: There was strong bony union of the fracture; there was cutaneous anæsthesia of the back of the hand, with œdema and glazed skin, complete paralysis of extensor and supinator muscles.

Under a diagnosis of compression of the nerve, an operation was suggested to the patient, who demurred. Faradism, as the next resort to exercise the muscles, was advised.

About this time the patient left the city and placed herself under the care of a specialist, who applied both galvanic and Faradic currents to the arm several times a week but without improvement.

Two months later the lady returned unimproved and ready for any measures which promised relief. Accordingly, the nerve was cut down upon and found just as it reached the front of the internal intermuscular septum; from this point it was traced backward through what had been the musculo-spiral groove, but now corresponded to the line of fracture and was filled with callus, against which the nerve was found compressed by a band of cicatricial tissue about an inch in width and one-fourth of an inch thick.

This cicatrix was the result of laceration of the soft parts, dependent upon the extreme displacement of fragments of fractured bone. The nerve was not caught in bony callus, but was found free in its sheath, though diminished to about one-third of its normal size, and tinted a yellowish brown at the point of constriction, this tint shading off into light yellow below and clear white above.

The cicatrix was excised and the cut triceps muscle united by catgut sutures, bone drainage-tube introduced, and the wound closed. The incision healed by first intention.

Within twenty-four hours after the operation acute pain appeared in the hand and forearm, but gradually subsided, lasting perhaps ten days.

Two weeks afterward very slight extension of the wrist could be accomplished, sensation having returned nearly to normal.

Improvement from this time on was constant, and twelve weeks after the operation the patient had resumed her former occupation—that of fancy embroidery, requiring a high degree of skill in the use of fingers, and was apparently as skillful as ever.

At the present date the hand and arm seem perfectly normal, with the exception of the scar left by the operation.

The points of greatest interest are, briefly:

In Case I, primary union of the nerve though the severed ends were left for twenty-four hours before being approximated.

In Case II, primary union and early recovery, although the circulation in the hand was not fully re-established for two days.

In Case III, after twelve weeks' entire loss of function, the nerve showed unmistakable signs of recovery within two weeks from the date of the operation, and in ten weeks more had entirely recovered.

4306 Lake Avenue.

